# C-Terminal Fragment of Agrin (CAF): A Novel Marker for Progression of Kidney Disease in Type 2 Diabetics

**DOI:** 10.1371/journal.pone.0143524

**Published:** 2015-12-02

**Authors:** Vasilios Devetzis, Arezoo Daryadel, Stefanos Roumeliotis, Marios Theodoridis, Carsten A. Wagner, Stefan Hettwer, Uyen Huynh-Do, Passadakis Ploumis, Spyridon Arampatzis

**Affiliations:** 1 Department of Nephrology, Hypertension and Clinical Pharmacology, Inselspital, Bern University Hospital, Bern, Switzerland; 2 Institute of Physiology, University of Zurich, Zurich, Switzerland; 3 Department of Nephrology, University Hospital of Alexandroupoli, Alexandroupoli, Greece; 4 Neurotune AG, Schlieren, Switzerland; Biomedical Research Foundation of the Academy of Athens, GREECE

## Abstract

**Background:**

Diabetes is the leading cause of CKD in the developed world. C-terminal fragment of agrin (CAF) is a novel kidney function and injury biomarker. We investigated whether serum CAF predicts progression of kidney disease in type 2 diabetics.

**Methods:**

Serum CAF levels were measured in 71 elderly patients with diabetic nephropathy using a newly developed commercial ELISA kit (Neurotune®). Estimated glomerular filtration rate (eGFR) and proteinuria in spot urine were assessed at baseline and after 12 months follow up. The presence of end stage renal disease (ESRD) was evaluated after 24 months follow-up. Correlation and logistic regression analyses were carried out to explore the associations of serum CAF levels with GFR, proteinuria, GFR loss and incident ESRD. Renal handling of CAF was tested in neurotrypsin-deficient mice injected with recombinant CAF.

**Results:**

We found a strong association of serum CAF levels with eGFR and a direct association with proteinuria both at baseline (r = 0.698, p<0.001 and r = 0. 287, p = 0.02) as well as after 12 months follow-up (r = 0.677, p<0.001 and r = 0.449, p<0.001), respectively. Furthermore, in multivariate analysis, serum CAF levels predicted eGFR decline at 12 months follow-up after adjusting for known risk factors (eGFR, baseline proteinuria) [OR (95%CI) = 4.2 (1.2–14.5), p = 0.024]. In mice, injected CAF was detected in endocytic vesicles of the proximal tubule.

**Conclusion:**

Serum CAF levels reflect renal function and are highly associated with eGFR and proteinuria at several time points. Serum CAF was able to predict subsequent loss of renal function irrespective of baseline proteinuria in diabetic nephropathy. CAF is likely removed from circulation by glomerular filtration and subsequent endocytosis in the proximal tubule. These findings may open new possibilities for clinical trial design, since serum CAF levels may be used as a selection tool to monitor kidney function in high-risk patients with diabetic nephropathy.

## Introduction

Chronic kidney disease (CKD) represents a global public health problem affecting more than 1 in 10 adults worldwide [1]. Diabetes is the leading cause of CKD in the developed world and people with both diabetes and chronic kidney disease have a greatly increased risk of all-cause mortality, cardiovascular mortality, and end-stage renal disease (ESRD) [2]. Although several factors have been identified to predict risk of developing progressive nephropathy in diabetic patient populations, none sufficiently predict the risk for individual patients [3].

Currently the simultaneous evaluation of albuminuria and glomerular filtration rate (GFR) is recommended by the Kidney Disease Improving Global Outcomes (KDIGO) guidelines for the prediction of progression in diabetic nephropathy [4]. A growing body of evidence challenges the traditional conceptual model of the course of diabetic nephropathy [5,6], since it can present with a rapid decline of renal function and no overt albuminuria or progressive proteinuria [7–10]. Based upon these clinical observations, more reliable biomarkers are urgently needed in the clinic to predict renal outcome in patients with early stages of CKD in diabetic nephropathy [11].

Agrin is the major heparin sulfate proteoglycan in the glomerular basement membrane and a ubiquitous component of the extracellular matrix [12,13]. Neurotrypsin, a serine protease, cleaves agrin at two distinct molecular sites generating a 110 kDa fragment (CAF110) at the alpha site, whereas cleavage at the beta site produces the 22 kDa C-terminal fragment (CAF22) [14]. In human urine, CAF22 can be detected, suggesting renal clearance for this small fragment [15,16]. Furthermore, serum CAF22 (sCAF) as kidney function biomarker has only recently been explored in septic patients and in renal transplant recipients [17,18]. Both studies indicate that the sCAF concentration is associated and comparable to established parameters of renal function such as creatinine and cystatin C.

However, to date, there are no clinical studies, which have investigated whether sCAF could serve as biomarker in clinical practice for diabetic nephropathy and no animal studies addressing the renal handling of sCAF. We hypothesize that rising sCAF levels may reflect progression of kidney damage and dysfunction. In this prospective study in a cohort of patients with diabetic nephropathy, we aimed to: 1) explore and validate the cross-sectional associations between sCAF and the currently used clinical markers of kidney damage and dysfunction; estimated glomerular filtration rate (eGFR) and proteinuria (protein to creatinine ratio [PCR]) 2) examine the independent predictive performance of sCAF for renal function decline and ESRD and 3) study the renal handling of CAF in neurotrypsin deficient mice lacking endogenous CAF22 production.

## Methods

### Study design and patient cohort

The present study was designed as a prospective observational cohort study. Study subjects were recruited from the outpatient clinic of Department of Nephrology, University Hospital of Alexandroupoli, Greece. Patients were recruited if they fulfilled the following inclusion criteria; (i) age > 18 years, (ii) ability to provide written, informed consent (iii) type 2 diabetes, defined as the use of oral glucose-lowering treatment, a fasting plasma glucose >7.0 mmol/l (126 mg/dl) or non-fasting plasma glucose >11.1 mmol/l (>200 mg/dl). The presence of diabetic nephropathy was defined by microalbuminuria [albumin to creatinine ratio 3–30 mg/mmol (30–300mg/g)] or persistent albuminuria [(albumin: creatinine ratio >30 mg/mmol (>300mg/g)] in three consecutive measurements in sterile spot urine sample during a 6-month period, presence of diabetic retinopathy, and no clinical or laboratory evidence of kidney or urinary tract disease [19]. Subjects were considered to have diabetic retinopathy if they showed nonproliferative or proliferative stages in fundoscopy through dilated pupils or had a history of retinal laser surgery (photocoagulation) for diabetic retinopathy.

The main exclusion criteria were clinical or laboratory evidence of non-diabetic nephropathy such as active malignancy, infection or autoimmune disease. The study was approved by the Ethics Committee of the Scientific Council of the University Hospital of Alexandroupoli, was in accordance with Helsinki Declaration of Human Rights and all subjects gave written informed consent.

The diagnosis and staging of CKD was assessed based on the Clinical Practice Guidelines from the National Kidney Foundation–Kidney Disease Outcomes Quality Initiative [20]. GFR was estimated (eGFR) using the Chronic Kidney Disease Epidemiology Collaboration equation (CKD-EPI), which is more accurate and less biased than the MDRD Study equation, especially in patients with higher GFR, resulting in reduced misclassification of CKD [21]. The onset of ESRD was defined as the date of first dialysis or transplantation.

### Biochemical analysis

After an overnight fasting of 8 h blood samples were drawn from a peripheral vein of the patients into vacutainer tubes without anticoagulant in order to obtain serum. Samples for fasting blood glucose levels, potassium, sodium, calcium, phosphate, protein, albumin, urea, creatinine and HbA1c were measured according to routine laboratory methods. Proteinuria was determined in spot urine by an automated biochemistry analyzer (Clinical Chemistry System ADVIA 2400, Siemens). Blood samples were immediately centrifuged at 4,000 rpm for 10 min at ambient temperature, and the extracted serum was aliquoted and stored at 253 K (-20°C) until use.

### C-terminal agrin fragments (CAF)

CAF levels were measured using the NTtotalCAF ELISA kit from Neurotune AG (www.neurotune.com). This assay detects both the large C-terminal agrin fragment CAF110 generated by alpha cleavage and the small C-terminal agrin fragment generated by beta cleavage (CAF22). The molecular ratio of both fragments is 1:5 (CAF110 vs CAF22). In brief, serum samples were diluted two-fold with sample incubation buffer and heated to 329 K (56°C) for 30 minutes. Samples were diluted 20 fold with dilution buffer. 100 μL of diluted sample was added to an ELISA plate pre-coated with a monoclonal catcher antibody raised against CAF. The plate was then incubated for 16 hours at room temperature. After rinsing 3 times with washing buffer, the monoclonal detector antibody was added for 30 minutes at room temperature. The plate was then washed as previously described [6]. Streptavidin- conjugated HRP was then added for 30 minutes at room temperature. After washing, TMB substrate was added and developed for 30 minutes. The reaction was then stopped with an acidic solution and the resulting absorbance was measured on an ELISA plate reader at 450 nm. The results were quantified using the calibration curve prepared independently on each individual plate. The lower detection limit was 40 pmol/L. Independent analysis revealed a mixed intra- and inter-assay %CV < 13% for serum samples.

### Statistical analysis

Variables were tested for normality using the Kolmogorov-Smirnov test. Comparisons between categorical variables were performed by the chi- square test or the Fisher’s exact test when appropriate. Differences in continuous variables between two groups were assessed using the Student’s t-test, the Mann-Whitney’s U-test or one-way ANOVA when appropriate. Pearson and Spearman correlation coefficients were calculated as appropriate between CAF or logarithmic transformed CAF (to achieve normal distribution), creatinine, GFR, proteinuria, age, BMI, HbA1c and duration of T2DM at baseline and at 12 months follow-up. Correlation analysis of decline of renal function (ΔGFR) and progression to ESRD with various risk factors was also conducted to determine possible outcome predictors.

Multiple logistic regression analysis was carried out to further explore the predictive role of serum CAF levels with GFR decline. The variables entered in the models were known risk factors associated with progression of kidney disease. For both outcomes a basic and an advanced model were used with the following variables: 1) Basic model A: CAF, baseline GFR and baseline proteinuria, 2) Advanced model B: CAF, baseline GFR, baseline proteinuria, renin-angiotensin-aldosterone system blockade, age and BMI; and 3) Advanced C: Serum creatinine, baseline GFR, baseline proteinuria, renin-angiotensin-aldosterone system blockade, age and BMI. Variables retained in all final models were chosen according to clinical significance. The dataset variables can be found in the supporting Information dataset (S1 Table). A two-sided p value < 0.05 was considered significant. The IBM SPSS Statistics 20.0 statistical software package (SPSS Insc, Chicago, Illinois, USA) was used for all calculations.

### Animal experiments

Experiments were performed in 8–10 weeks old male neurotrypsin knockout mice (NT KO). The generation and genotyping of neurotrypsin KO mice has been previously described [22]. All animal experiments were conducted according to Swiss laws for the welfare of animals and were approved by the local Zurich Veterinary Authority (Kantonales Veterinäramt Zürich). The animals had free access to food and tap water.

Mice were pretreated 60 min before experiments with an injection of leupeptin (5 mg/mouse, Sigma Aldrich, Buchs, Switzerland), an inhibitor of lysosomal degradation. Wildtype and neurotrypsin KO mice were anesthetized by i.p. injection of xylazin and ketamin and were injected with 100 ml of a mixture containing recombinant hCAF22 (100 ng/mouse, produced by Neurotune) dissolved in 0.9% NaCl and FITC-labeled sinistrin (100 ng/ mouse). FITC-sinistrin is cleared from circulation exclusively by glomerular filtration and served as control for successful injection. Before injection of hCAF, the urinary bladder was completely emptied through a small abdominal incision to allow collection of urine produced during the experimental period. Mice received also a bolus i.p. of 0.5 ml of 25 mM NaHCO_3_/150 mM NaCl to prevent dehydration and to promote diuresis. Mice were kept warm at 37°C by placing animals on a heating tablet for the rest of the experiment. Twenty or 60 minutes after hCFA injections, mice perfused with PBS and PLP (see below) through the heart to obtain fixed kidneys for later immunohistochemistry and localization of recombinant hCAF.

### Immunohistochemistry

Mice were anesthetized with ketamine/xylazine (i.p.) and systemically perfused through the left ventricle with phosphate-buffered saline (PBS) to remove blood followed by paraformaldehyde-lysine-periodate (PLP) fixative (50 ml/mouse) [23]. Kidneys were removed and fixed overnight at 4°C by immersion in PLP. Kidneys were washed 3 times with PBS and 5 μm cryosections were cut after cryoprotection with 2.3 M sucrose in PBS for at least 12 h. Immunostaining was carried out as described previously [24]. Briefly, sections were shortly incubated in microwave with Tris-HC [pH 10], following 1% (wt/vol) SDS for 5 min for retrieval of antigenic sites, washed 3 times with PBS and incubated with PBS containing 1% bovine serum albumin for 15 min prior to the primary antibody. The primary antibodies were mouse monoclonal anti cleaved Agrin Abs (CAF; 14B7B8; 1:1000) and (CAF; 12A11D11; 1:1000) (provided by Neurotune diluted in PBS and applied overnight at 4°C. Sections were then washed twice for 5 min with high NaCl PBS (PBS + 18 g NaCl/l), once with PBS, and incubated with dilutions of the secondary antibodies (donkey anti-rabbit 594 (1:500), donkey anti-mouse 488 (1:200), donkey anti-mouse Alexa 594 (1:500), donkey anti-rabbit Alexa 488 (1:500) (Molecular Probes, Oregon, USA) and from DAPI (1 mg/ml) (1:500) for 1 h at room temperature. Sections were again washed twice with high NaCl PBS and once with PBS before mounting with VectaMount (Vector Laboratories, Burlingame, CA). Sections were viewed with a Zeiss LSM 410 confocal microscope or a Leica DFC490 charged-coupled device camera attached to a Leica DM 6000 fluorescence microscope (Leica, Wetzlar, Germany). Images were processed (overlays) using Adobe Photoshop.

## Results

### Study population

A total of 71 consecutive patients with long standing (>10 years) diabetic nephropathy, were recruited from January 2010 to December 2014. A full medical history and physical examination was performed by a trained physician at baseline (time-point 0; T0) and after 12 months (time-point 1; T1). All patients were of Caucasian origin. Demographic, clinical and laboratory data of all enrolled patients at T0 and T1 as well as of groups of progressors (loss of GFR > 1ml/min/1.73m^2^) and non progressors (stable GFR) are given in Table 1.

**Table 1:** Anthropometric, clinical and biochemical characteristics of patients with TD2M and CKD with stable (or increased) and with decreased GFR at one year follow-up.

	All patients (N = 71)	Stable GFR (N = 31)	Decreased GFR (N = 40)	P-value^a^
Age (years)	70.9 (8.8)	71.8 (6.4)	70.2 (10.3)	0.79
Gender (M/F)	36/35	11/20	25/15	0.024*
BMI (kg/m2)	32.4 (5.9)	33.0 (5.9)	31.9 (6.0)	0.49
Duration of T2DM (years)	17.4 (9.7)	16.5 (7.6)	18.1 (11.2)	0.83
RAAS blockade (%)	49 (69)	25 (80.6)	24 (60)	0.06
Urea (mg/dl)	13.1 (6.76)	10.5 (3.98)	15.1 (7.77)	0.004*
Potassium (mEq/L)	4.8 (0.5)	4.7 (0.3)	4.9 (0.6)	0.34
Sodium (mEq/L)	138.3 (3.0)	138.2 (3.0)	138.4 (3.0)	0.71
Calcium (mmol/L)	2.35 (0.15)	2.38 (0.13)	2.33 (0.18)	0.35
Phosphate (mmol/L)	1.23 (0.26)	1.16 (0.19)	1.29 (0.29)	0.03*
Protein (g/L)	73 (6)	74 (07)	72 (6)	0.34
Albumin (g/L)	43 (4)	44 (3)	42 (4)	0.06
HbA1c (%)	8.6 (9.3)	7.5 (1.3)	9.5 (12.3)	0.88
Crea T0 (mg/dl)	123.8 (53)	114.9 (35.4)	132.6 (61.9)	0.19
Crea T1 (mg/dl)	123.8 (79.6)	106.1 (35.4)	150.3 (79.6)	<0.001*
eGFR T0 (ml/min/1.73 m2)	43 (27)	44 (23)	40.5 (30.5)	0.62
eGFR T1 (ml/min/1.73 m2)	43 (27)	54 (24)	34 (23)	<0.001*
ΔGFR T0-T1 (ml/min/1.73 m2)	-2.0 (11)	5.0 (8)	-6.0 (8)	<0.001*
PU T0 (mg/24h)	270 (600)	245 (430)	370 (705)	0.28
PU T1 (mg/24h)	300 (770)	240 (490)	465 (985)	0.059
ΔPU T0-T1 (mg/24h)	10 (270)	0 (200)	45 (390)	0.19
ESRD (%)	6 (8.5)	0 (0)	6 (15)	0.024*
Total CAF (pmol/L)	1091.4 (890.2)	993.4 (737.8)	1145.9 (1571.1)	0.09

Continuous variables are presented as mean (S.D.) or median (interquartile range, IQR).

^a^ *P* values of Mann-Whitney *U* or one-way ANOVA test for differences of variables among patients with stable or decreased eGFR at one year follow-up.

* Statistical significance at the 0.05 level (two-tailed).

BMI, body mass index; HbA1c, glycosylated hemoglobin A1c; RAAS blockade, use of medicines affecting renin–angiotensin system; Crea T0, serum creatinine levels at timepoint 0 (baseline); Crea T1, serum creatinine levels at timepoint 1 (12 months); eGFR T0, estimated GFR assessed by the CKD-EPI formula at timepoint 0 (baseline); eGFR T1, estimated GFR assessed by the CKD-EPI formula at timepoint 1 (12 months); PU T0, Proteinuria assessed by protein to creatinine ratio at timepoint 0 (baseline); PU T1, Proteinuria assessed by protein to creatinine ratio at timepoint 1 (12 months); ΔGFR T0-T1, Algebric difference between eGFR at timepoint 1 and eGFR at timepoint 0; ΔPU T0-T1, Algebric difference between Proteinuria at timepoint 1 (12 months) and Proteinuria at timepoint 0 (baseline); ESRD, progression to end stage renal disease; Total-CAF, serum levels of C-terminal agrin fragment; Decreased GFR, patients with loss of eGFR ≥ 1 ml/min/1.73m^2^ during the 12 month follow-up.

There was no significant difference in age, BMI and duration of T2DM across the categories of patients with and without GFR decline. Urea and phosphate concentrations were significantly different across groups and as expected, increased in the group of patients with decreased GFR (p = 0.004, 0.03 respectively). There were significantly more males than females among GFR decliners compared with those with stable or increased GFR (62,5% vs 35,4%, p = 0.024). sCAF levels showed a trend towards higher values in those with deterioration of renal function (p = 0.09).

### Correlation analysis

**Fig 1:**
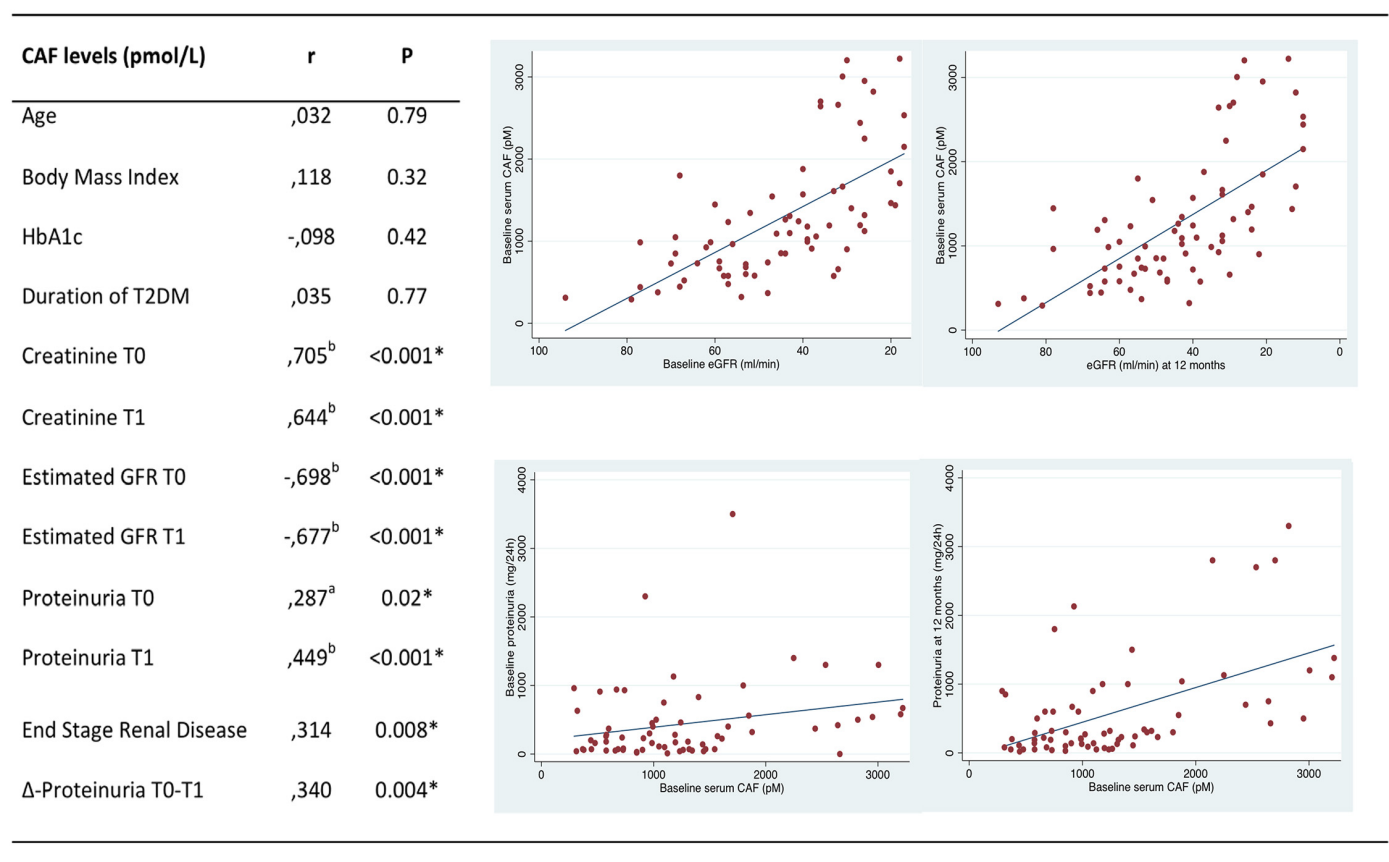
Correlation analysis of CAF levels with variables of laboratory and clinical significance and scatterplots of CAF with GFR and proteinuria. Values represent Spearman’s correlation coefficients. ^a^ Correlation is significant at the 0.05 level (2-tailed). HbA1c, glycosylated hemoglobin A1c; T0, Time point 0 (Baseline); T1, Time point 2 (12 months); Δ-Proteinuria T0-T1, Increase of proteinuria (≥500mg/day) during the first year of follow-up.

Correlation matrix analysis showed that values of creatinine and eGFR at both time-point 0 and time-point 1 were significantly associated with sCAF levels (p<0.001) (Fig 1). sCAF concentration was also correlated with proteinuria at baseline and at T1 (p = 0.02 and p<0.001, respectively) (Fig 1). Furthermore, sCAF levels were strongly correlated with progression to ESRD (r = 0.314, p = 0.008) (Fig 1). At 24 months follow-up (time point 2; T2) 6 of the 71 patients progressed to ESRD (8.5%). Baseline sCAF levels were significantly elevated in those who reached ESRD compared to the group of patients that did not (p = 0.009). sCAF also showed a significant association with the rapid increase of proteinuria (>500 mg/day) at T1 (r = 0.340, p = 0.004) (Fig 1). The baseline sCAF level was higher in those who presented a rapid increase of proteinuria than in patients with stable, decreased or mild increase (<500 mg/day) of proteinuria (p = 0.005). A significant positive association was observed between rapid increase of proteinuria and progression to ESRD (x^2^, p = 0.005).

### Logistic regression analysis

Logistic regression models of the association of renal outcomes (defined as GFR decline > 1ml/min1.73m^2^) with baseline sCAF levels and other clinically significant regressors, assessed by 2 complementary models: Basic model A (CAF, baseline GFR and baseline proteinuria) and advanced model B (CAF, baseline GFR, RAAS-Blockade, baseline proteinuria, age, BMI) are shown in Table 2. CAF was the only variable which was significantly associated with GFR loss (≥ 1 ml/min/1.73m^2^) in both model A [OR (95%CI) = 4.2 (1.2–14.5), p = 0.024] and model B [OR (CI) 4.15 (1.14–15.07), p = 0.031]. Performing a logistic regression analysis based on the advanced model including serum creatinin (model C) resulted in a non-significantly associated OR concerning GFR loss compared to CAF (p = 0.079).

**Table 2:** Association of GFR-decline with serum CAF and additional clinical regressors.

		GFR decline at 1 year ≥ 1 ml/min/1.73m^2^		
		OR^a^	CI^b^	P-value
**Model A**	logCAF	4.18	1.2–14.5	0.024*
	eGFR T0	1.03	0.99–1.08	0.102
	PU T0	1.0002	0.99–1.0006	0.299
**Model B**	logCAF	4.15	1.14–15.07	0.031*
	eGFR T0	1.03	0.99–1.08	0.109
	RAAS-Block	0.3	0.09–1.06	0.064
	PU T0	1.0002	0.99–1.0006	0.321
	Age	1.001	0.93–1.07	0.964
	BMI	0.97	0.89–1.07	0.438
**Model C**	SCreat T0	6.3	0.8–50.29	0.079
	eGFR T0	1.05	0.98–1.12	0.104
	RAAS-Block	0.4	0.12–1.5	0.202
	PU T0	1.0002	0.99–1.0006	0.348
	Age	1.020	0.95–1.009	0.554
	BMI	0.99	0.90–1.08	0.321

^a^ OR, odds ratio

^b^ CI, 95% confidence interval

* Significance levels at 0.05

BMI, body mass index; eGFR T0, estimated GFR assessed by the CKD-EPI formula at baseline; PU T0, Proteinuria assessed by protein to creatinine ratio at baseline; logCAF, serum CAF levels (log-transformed). GFR, estimated glomerular filtration rate; SCreat, Serum creatinine; renin-angiotensin-aldosterone system blockade (RAAS-Block).

### Animal experiments

**Fig 2:**
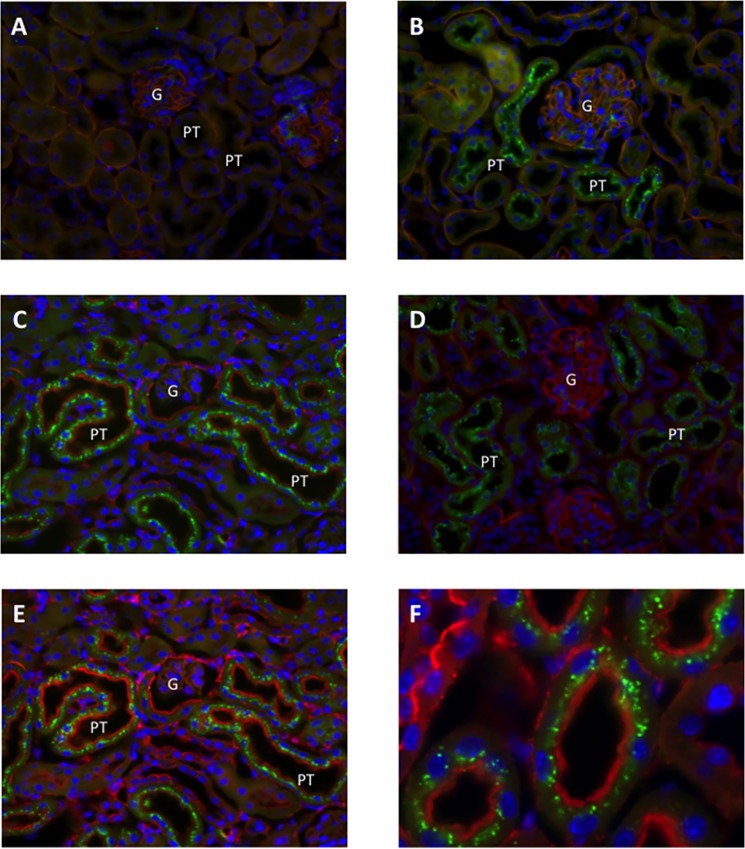
Accumulation of recombinant hCAF22 in mouse proximal tubule. Mice were injected with saline or hCAF22 and all sections stained in parallel with the antibodies as indicated. A. Localization of full length agrin (red) and hCAF22 (green) in NT KO kidney injected with saline. B. Localization of full length agrin (red) and hCAF22 (green) in NT KO mouse kidney 20 min after hCAF22 injections. C. Localizations of hCFA22 (green) and actin (red) in NT KO mouse kidney 20 min after hCAF22 injections. D. Localization of full length agrin (red) and hCAF22 (green) in NT KO mouse kidney 60 min after hCAF22 injections. E-F. Localizations of hCFA22 (green) and actin (red) in NT KO mouse kidney 60 min after hCAF22 injections. A- E Original magnifications between 400–630 x, for F original magnification 1000 x.

In order to test for the role of the kidney in determining serum CAF levels, we injected recombinant human CAF22 into neurotrypsin KO mice. These mice lack endogenous CAF22 due to the absence of the specific protease required for the cleavage of agrin [22]. In kidneys from NT KO mice injected with saline, no recombinant hCAF22 could be observed providing evidence for the specificity of the antibody. However, full length agrin was detected in the glomerulum and in the basement membrane of all tubules (Fig 2A). Twenty and sixty minutes after hCFA22 injections, hCAF22 accumulated in subapical vesicles along the proximal tubule consistent with the reabsorption of hCFA22 from urine via apical endocytosis (Fig 2B–2F).

## Discussion

The major findings in this prospective study of predominantly elderly patients with long-term diabetic nephropathy are that sCAF levels are strongly associated with eGFR and proteinuria. Of note these associations were preserved and evident in subsequent measurements for both parameters at T1. Higher baseline sCAF levels were significantly correlated with progression to ESRD and notably elevated in subjects with an overt increase of proteinuria at T1. Furthermore, sCAF levels predicted loss of renal function, as mirrored by eGFR decline at T1 even after adjusting for multiple well established risk factors, and in particular, for baseline GFR and proteinuria.

Our results are consistent with previous studies that have reported associations between sCAF levels and renal function. Steubl et al. showed that sCAF was highly correlated with kidney function in a renal transplant cohort [18]. Additionally, Drey and colleagues demonstrated that sCAF levels are strongly associated and comparable to established renal function parameters (creatinine and cystatin C) in critical ill patients [17]. In the present study, serum levels of CAF at baseline were significantly higher in diabetics, which progressed to ESRD. Importantly, the association between sCAF levels and the different aspects of kidney dysfunction were also evident irrespective of proteinuria. Although sCAF reflect renal function and is highly associated with eGFR and proteinuria at several time points, it predicted loss of renal function also in the absence of proteinuria in diabetic nephropathy. sCAF seems to overcome a significant prognostic restriction of proteinuria in a subset of patients with rapid decline of renal function and no progressive proteinuria, a finding that to our knowledge has not been reported before.

Our animal experiments are consistent with the hypothesis that CAF22 is cleared from the circulation by glomerular filtration. Injected human recombinant hCAF22 accumulated in subapical vesicles along the proximal tubule. These vesicles likely represent endocytic vesicles containing low molecular weight proteins that escaped the glomerular filtration barrier. CAF22 has only 22 kDa size and is expected to be filtered to a large extent similar to other proteins of this size [25]. The subapical localization of vesicles containing hCAF22 strongly suggests that hCAF22 is taken up from urine by endocytosis, possibly receptor-mediated endocytosis. It is unlikely that hCAF22 is secreted into urine by proximal tubules in quantitatively relevant amounts since we never detected hCAF22 positive vesicles close to the basolateral membrane. Reabsorption of hCAF22 along with other low molecular weight proteins will determine its urinary excretion. Thus, reduced glomerular filtration in kidney disease would increase serum CAF22 levels whereas a selective reduction in proximal tubular reabsorption may increase urinary CAF22 levels. While the exact mechanism of CAF production and trafficking in the kidney is currently under investigation, our findings further support the notion that CAF may reflect both structural (proteinuria) and functional (glomerular filtration rate) alterations.

CKD has a major public impact worldwide, with a global prevalence of 10% and diabetic nephropathy is the primary reason of CKD [1,26,27]. sCAF may represent a promising biomarker of kidney damage and progression to ESRD in diabetic nephropathy. However, more studies with sufficient follow up are needed to evaluate the clinical value of sCAF measurements for: 1) the detection of kidney damage, 2) the prediction of GFR decline and 3) the development of ESRD. The correlation between sCAF levels and the progression of kidney dysfunction in other cohorts [28] as well as in the present study indicates that sCAF measurements may be of clinical importance. This finding may open new possibilities for clinical trial design, since sCAF levels could be used as a selection tool for high-risk patients (individuals with CKD and in particular with diabetic nephropathy and no overt proteinuria at baseline) and for monitoring treatment effects in diabetic nephropathy.

Limitations of the present study include the single center design, the short-term follow-up and unknown cross-relevance to other age and ethnic groups. While a multitude of relevant variables were adjusted in our multivariate analyses, as with all observational studies, it is possible that unmeasured confounders may have influenced our estimates and results. No conclusions regarding causality should be drawn from our prospective observational data. However, the strong associations with eGFR and proteinuria in diabetics are of interest because sCAF has been shown to be a prognostic marker of kidney dysfunction in other cohorts including patients with CKD and in critically ill patients with acute kidney injury [17,18].

Our findings suggest that sCAF, a novel kidney function and injury biomarker, is associated with subsequent renal function loss irrespective of proteinuria at baseline. The association we observed was strong and was not altered by adjusting for several risk factors. sCAF levels provide important information on the long-term outcome of patients with diabetic nephropathy, which exceed a simple reflection of glomerular filtration rate and proteinuria. sCAF measurements may have a substantial impact on clinical trial design as a selection tool since it holds the potential to identify individuals with the higher progression risk for diabetic nephropathy. Further studies are needed in order to replicate our results and to confirm sCAF as a monitoring tool for rapid disease progression in diabetic kidney disease.

## Supporting Information

S1 Table Supporting Information dataset. Dataset with available variables as excel file and codebook.
(XLSX)
